# Astragalus polysaccharide restores autophagic flux and improves cardiomyocyte function in doxorubicin-induced cardiotoxicity

**DOI:** 10.18632/oncotarget.13596

**Published:** 2016-11-25

**Authors:** Yuan Cao, Tao Shen, Xiuqing Huang, Yajun Lin, Beidong Chen, Jing Pang, Guoping Li, Que Wang, Sylvia Zohrabian, Chao Duan, Yang Ruan, Yong Man, Shu Wang, Jian Li

**Affiliations:** ^1^ Peking University Fifth School of Clinical Medicine, The MOH Key Laboratory of Geriatrics, Beijing Hospital, National Center of Gerontology, Beijing, 100730, China; ^2^ Department of Cardiology, Boston Children's Hospital, Enders 1250, Boston, MA, 02115, US; ^3^ Beijing Key Laboratory of Plant Resources Research and Development, Beijing Technology and Business University, Beijing 100037, China; ^4^ Beijing Anzhen Hospital, Capital Medical University and Beijing Institute of Heart, Lung and Blood Vessel Diseases, Beijing, 100029, China

**Keywords:** astragalus polysaccharide, doxorubicin, cardiomyocyte, autophagy, AMPK/mTOR

## Abstract

Doxorubicin (adriamycin), an anthracycline antibiotic, is commonly used to treat many types of solid and hematological malignancies. Unfortunately, clinical usage of doxorubicin is limited due to the associated acute and chronic cardiotoxicity. Previous studies demonstrated that Astragalus polysaccharide (APS), the extracts of *Astragalus membranaceus*, had strong anti-tumor activities and anti-inflammatory effects. However, whether APS could mitigate chemotherapy-induced cardiotoxicity is unclear thus far. We used a doxorubicin-induced neonatal rat cardiomyocyte injury model and a mouse heart failure model to explore the function of APS. GFP-LC3 adenovirus-mediated autophagic vesicle assays, GFP and RFP tandemly tagged LC3 (tfLC3) assays and Western blot analyses were performed to analyze the cell function and cell signaling changes following APS treatment in cardiomyocytes. First, doxorubicin treatment led to C57BL/6J mouse heart failure and increased cardiomyocyte apoptosis, with a disturbed cell autophagic flux. Second, APS restored autophagy in doxorubicin-treated primary neonatal rat ventricular myocytes and in the doxorubicin-induced heart failure mouse model. Third, APS attenuated doxorubicin-induced heart injury by regulating the AMPK/mTOR pathway. The mTOR inhibitor rapamycin significantly abrogated the protective effect of APS. These results suggest that doxorubicin could induce heart failure by disturbing cardiomyocyte autophagic flux, which may cause excessive cell apoptosis. APS could restore normal autophagic flux, ameliorating doxorubicin-induced cardiotoxicity by regulating the AMPK/mTOR pathway.

## INTRODUCTION

Doxorubicin (DOX) is widely used as a broad-spectrum anticancer chemotherapeutic drug. Treatment with this drug results in generation of reaction oxygen species (ROS), DNA mutagenesis, inhibition of protein synthesis, cell membrane damage, and cell apoptosis [1]. Although DOX is beneficial as a cancer therapeutic medicine, it is also associated with acute and chronic cardiotoxicity, which leads to cardiomyopathy and congestive heart failure [2–4]. Elucidating the mechanism underlying DOX-induced cardiotoxicity may identify strategies to reduce cardiomyopathy risks for cancer patients.

Previous studies suggested that free radical-induced mitochondrial damage due to elevated ROS production is the major contributing factor to doxorubicin-induced cardiotoxicity, but recent reports have demonstrated that ROS scavengers failed to prevent heart failure [5, 6]. Therefore, there must be another mechanism regulating doxorubicin-induced cardiotoxicity. Typically, cardiotoxicity is attributed to cardiomyocyte loss through apoptosis and/or necrosis [7, 8]. However, recent evidence suggests that dysregulation of autophagy may also contribute to cardiomyocyte loss, causing heart failure [9].

Autophagy is an important cytoplasmic quality control system that removes protein aggregates and damaged organelles [15–16]. In physiological conditions, autophagy sustains optimal cardiomyocyte conditions by degrading or recycling misfolded proteins and damaged organelles [10–12]. Autophagic dysfunction may result in accumulation of misfolded/damaged proteins, inadvertently disrupting cardiac homeostasis. Alterations in autophagic homeostasis have been observed in various heart pathological conditions, such as oxidative stress or ischemia reperfusion [13–15]. However, the role of autophagy in doxorubicin-induced cardiomyopathy still remains poorly defined.

Astragalus species derived from the dry roots of Astragalus membranaceus [16] have been widely used in traditional Chinese medicine as an antiperspirant, antihypertensive, diuretic, and tonic treatments [17–19]. Astragalus polysaccharide (APS), the extracts of Astragalus membranaceus, also has strong anti-tumor and anti-glomerulonephritis activities [20, 21] and alleviates arterial inflammation [22]. APS has been shown to ameliorate the side effects of chemotherapeutic drugs in cancer patients. In a previous study, APS suppressed oxidative stress and apoptosis by regulating the PI3k/Akt and p38MAPK pathways [23].

In this study, we investigated the protective effects of APS in doxorubicin-induced cardiotoxicity, and provided novel insight into how APS regulate cardiomyocyte autophagy *in vivo* and *in vitro*. Our results revealed that APS could restore normal autophagic flux and improve heart function by regulating AMPK/mTOR pathway in doxorubicin-induced cardiotoxicity. These findings provide insight into the mechanisms of doxorubicin-induced cardiotoxicity in cancer patients and a new potential treatment for the side effects of doxorubicin.

## RESULTS

### Doxorubicin disturbs cardiomyocyte autophagic flux

**Figure 1:**
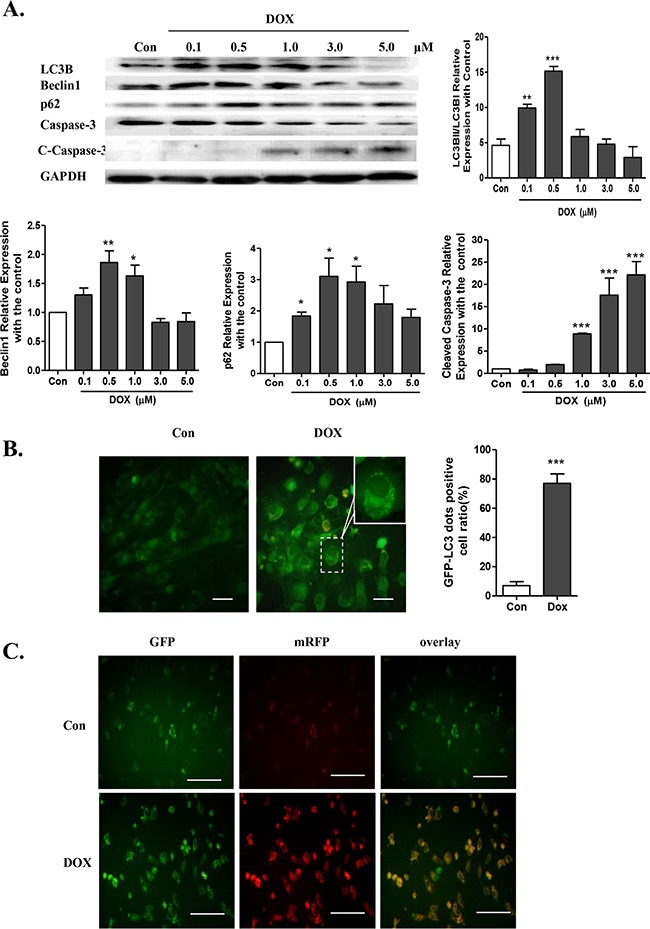
Doxorubicin induces cardiotoxicity by disturbing normal autophagy in cardiomyocytes **A.** LC3B I/II expression, caspase-3, cleaved caspase-3 (c-caspase-3), Beclin1 and p62/SQSTM1, as assayed by Western blots, in NRVMs treated with doxorubicin at 0.1-5.0 μM for 24 h (n = 5). **B.** Cardiomyocytes were transfected with GFP-LC3 adenovirus for 24 h prior to doxorubicin treatment to label autophagic vesicles. An M.O.I. of 30 was used. The transfection efficiency of the GFP-LC3 adenovirus after 24 h was above 95% (scale bar: 100 μM). **C.** Co-localization efficiency of mRFP with GFP signals of tfLC3 puncta in control (Con) and 0.5 μM doxorubicin-treated H9c2 cells (n = 4, scale bar: 50 μM). Transfection efficiency of tfLC3 after 24 h was approximately 70% (*P <0.05, **P< 0.01, ***P <0.001 vs. Con).

Our previous study revealed that doxorubicin treatment significantly reduced cell viability in a dose-dependent manner compared with the control, as shown by the cell viability assay [23]. To determine whether autophagy is involved in doxorubicin-induced cardiotoxicity, several important signaling pathway proteins were measured. The active (cleaved) caspase-3 (c-caspase-3) was significantly increased in a concentration-dependent manner in NRVMs treated with doxorubicin for 24 h, indicating elevated apoptosis (Figure 1A). Additionally, the level of LC3B II/I was significantly increased in 0.1 μM-0.5 μM doxorubicin treatment group, and was subsequently decreased in the 1 μM-5 μM doxorubicin-treated NRVMs (Figure 1A). So the cell viability decline might result from autophagy more than apoptosis in the treatment of 0.5μM doxorubicin. Therefore, 0.5 μM doxorubicin was chosen to establish the *in vitro* doxorubicin-induced cardiotoxicity model.

To determine whether doxorubicin-induced accumulation of LC3-II is caused by enhanced autophagic flux or impaired degradation, GFP-LC3 adenovirus, which indicates the formation of autophagosomes, was transduced into NRVMs. After the doxorubicin treatment, GFP signals were significantly elevated in NRVMs, indicating increased formation of autophagosomes (Figure 1B). Additionally, a marker for autophagic flux P62/SQSTM1, which is a polyubiquitin-binding protein that is degraded by autophagy and inversely related to autophagy during normal flux, was assessed by Western blot. We found that p62/SQSTM1 was significantly elevated in the NRVMs treated with doxorubicin, indicating an inability to complete autophagy (Figure 1A).

To assess autophagic flux, H9c2 cells were transiently transfected with a plasmid harboring a tandem fluorescent mRFP-GFP-LC3 (tfLC3) (Figure 1C). The GFP signal represents the autophagosomes, and the RFP signal represents the normal maturation of autophagosomes into autolysosomes. However, the GFP signal is not present in the acid environment of autolysosomes. Therefore, if most puncta exhibit both red and green signals, it indicates that autophagy is impaired at some steps. Under the dosage of 0.5 μM doxorubicin treatment, most of the red puncta were colocalized with green puncta (shown by the yellow signal in merged photos), indicating an impairment of autophagic flux. Together, the data suggested that the excess autophagy might be due to enhanced autophagosome formation as well as impaired autophagic flux.

### APS restores autophagy in doxorubicin-treated primary neonatal rat ventricular myocytes

**Figure 2:**
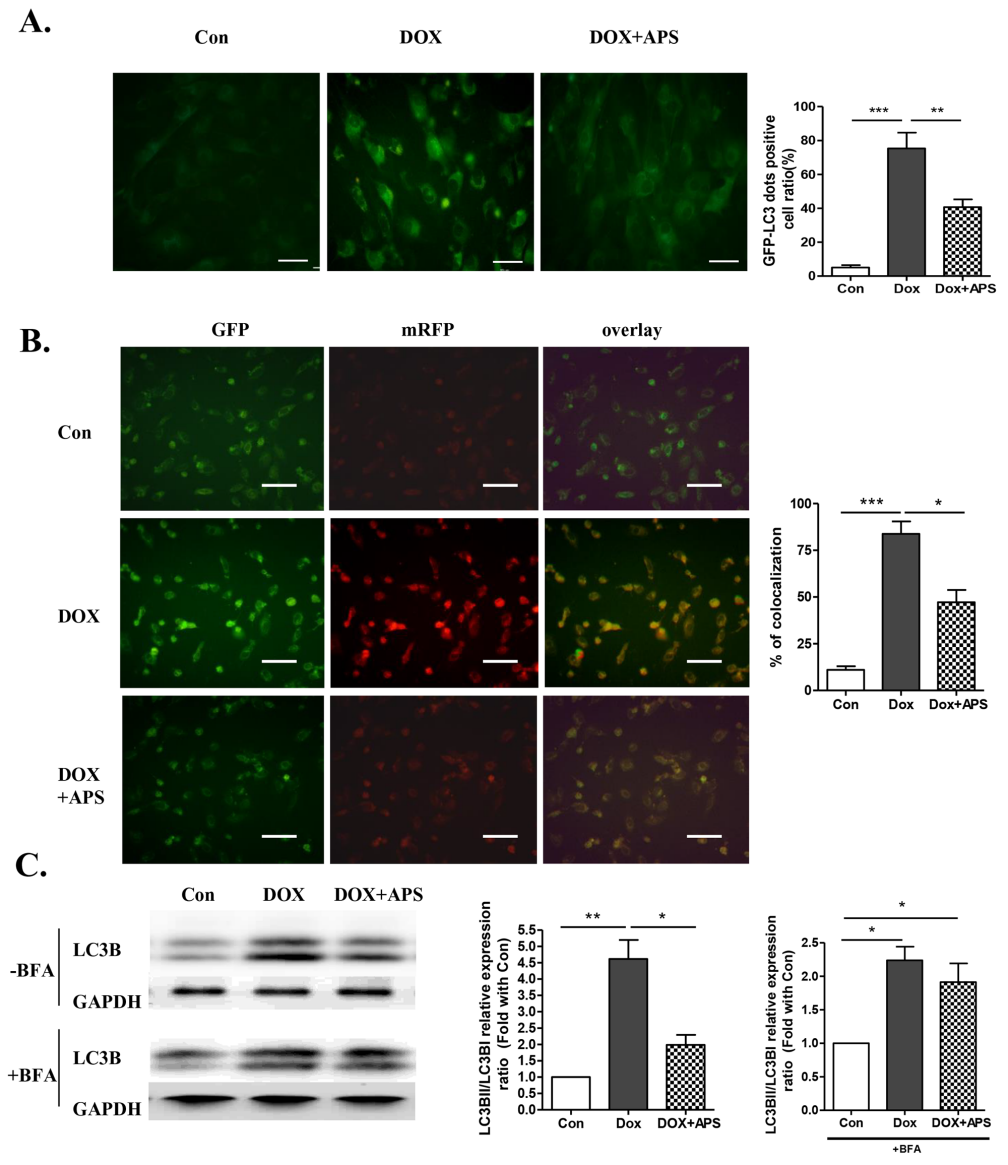
APS restores autophagy in doxorubicin-treated cardiomyocytes **A.** Primary neonatal rat ventricular myocytes were transfected with GFP-LC3 adenovirus for 24 h prior to doxorubicin and APS treatment (scale bar: 100 μM), GFP-LC3-positive dots were measured by ImageJ software (n = 4, scale bar: 100 μM). **B.** H9c2 cells were transfected with a plasmid expressing mRFP-GFP-LC3 for 24 h prior to doxorubicin and APS treatment. The cells were viewed and imaged with an inverted fluorescence microscope. Co-localization efficiency of mRFP with GFP signals of tfLC3 puncta was measured using ImageJ software, and the percentage of total number of GFP puncta is shown (n = 4, scale bar: 50 μM). Transfection efficiency of tfLC3 after 24 h was approximately 70% **C.** LC3B expression in Con, DOX-treated and DOX+APS-treated H9c2 cells with or without bafilomycin (BFA) pretreatment (n = 5). (*P<0.05, **P < 0.01).

In the presence of 50 μg/ml APS, autophagosome formation in cardiomyocytes was reduced compared with the 0.5 μM doxorubicin-treated group, as shown by the GFP-LC3B adenovirus assays (Figure 2A). As shown in Figure 2B, pretreatment with APS reversed the autophagic flux induced by doxorubicin, as shown by decreased co-localization of the tandem RFP-GFP-LC3 plasmid in merged images, which indicated that APS could attenuate doxorubicin-induced impaired autophagosome degradation. To further examine APS involvement in regulating autophagosome degradation, bafilomycin (BFA) was administered to block autophagic flux in cardiomyocytes. BFA is a classical inhibitor of the fusion of autophagosomes to lysosomes, which abolishes autophagosome degradation. As shown in Figure 2C, without BFA, the doxorubicin-induced LC3BII/I increase was alleviated by APS; following administration of BFA, which blocked the degradation of autophagosomes, LC3BII/I was still significantly increased in the doxorubicin group compared with the control. These data further confirmed that doxorubicin increased the formation of autophagosomes. However, compared with the doxorubicin group, APS treatment did not further decrease LC3B II/I. Therefore, we hypothesized that APS also played a role in restoring autophagosome degradation. Taken together, APS normalized autophagic flux by suppressing autophagosome formation and enhancing autophagosome degradation.

**Figure 3:**
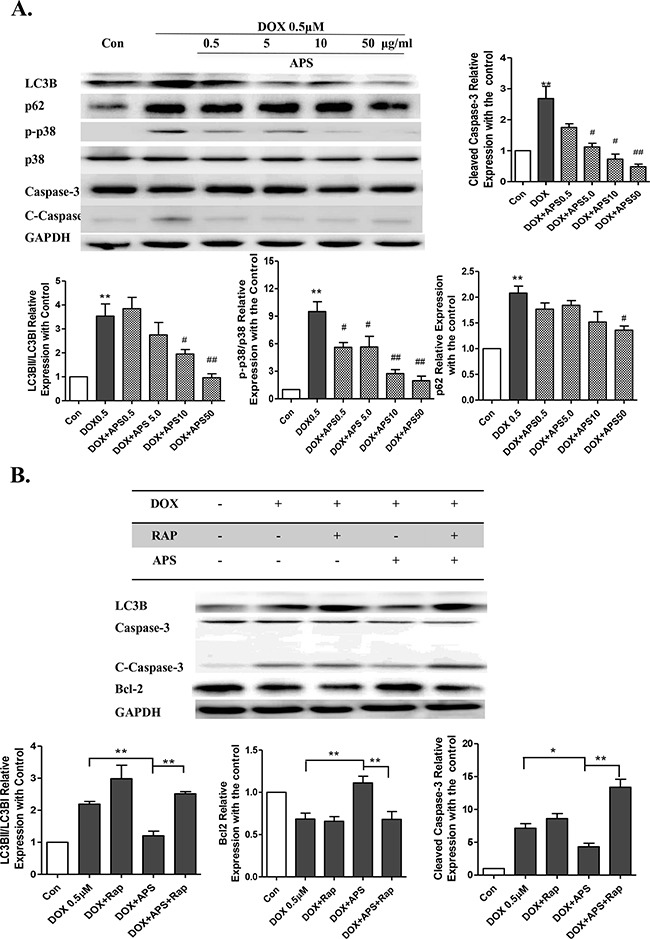
The protective role of APS in doxorubicin-induced cardiomyocyte injury **A.** APS reversed doxorubicin-induced increased LC3B II/I and p62/SQSTM1 and activated of caspase-3 in a concentration-dependent manner in NRVMs, as assayed by Western blots (n = 5, *P < 0.05, **P<0.01 vs. Con group; # P< 0.05,## P<0.01 vs. DOX group). **B.** Pretreatment of 100 nM rapamycin effectively reversed the APS protective effects in cardiomyocytes, as assessed by Western blots. The NRVMs were pretreated for 1 h with 100 nM rapamycin before APS (50 μg/ml) and DOX (0.5 μM) treatment. (n = 5, *P < 0.05, **P<0.01)

Moreover, as shown in Figure 3A, treatment with 50 μg/ml APS reversed the doxorubicin-induced increase in LC3B II/I and p62/SQSTM1. And caspase-3 activation was apparently suppressed by 50 μg/ml APS. These results indicate that excessive autophagy induced by doxorubicin promotes cardiomyocyte apoptosis (Figure 3A), while APS could potentially restore doxorubicin-induced excess accumulation of autophagosomes in cultured NRVMs. To confirm that APS could protect cardiomyocytes by attenuating LC3B activation, rapamycin (mTOR inhibitor), a classical autophagy activator, was used in cardiomyocyte. Pretreatment with 100 nM rapamycin effectively reversed the protective effect of APS in cardiomyocytes (Figure 3B). Activated caspase-3 was elevated in the APS protective group with pretreatment of rapamycin.

### APS ameliorates doxorubicin-induced heart failure by restoring normal autophagy and decreasing apoptosis *in vivo*

**Figure 4:**
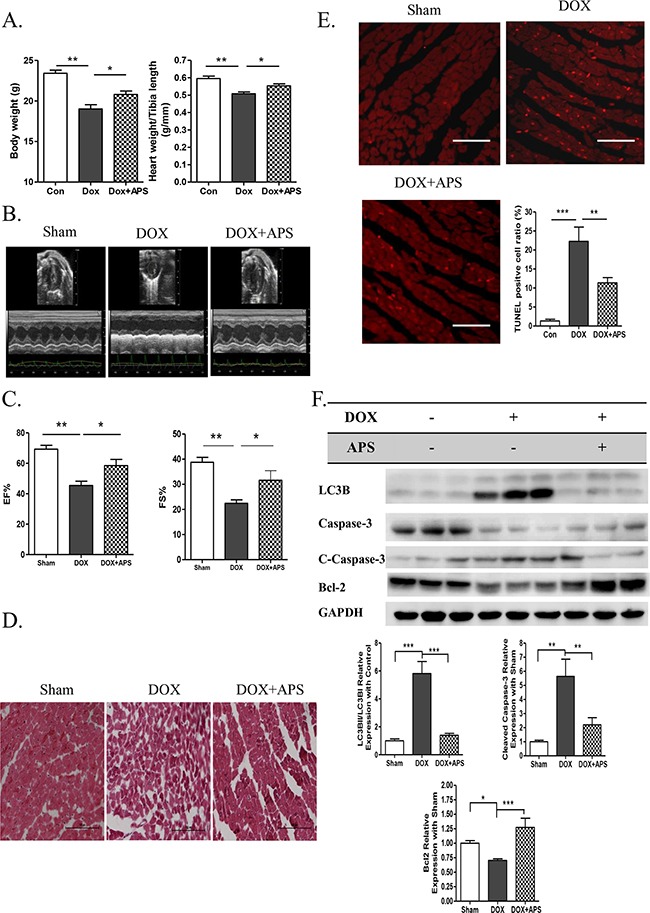
APS ameliorates doxorubicin-induced heart failure by restoring normal autophagy and decreasing apoptosis *in vivo* **A.** The average data of mouse body weight and heart weight/tibia length rate (g/mm) in sham, DOX, and DOX + APS groups 5 days after doxorubicin treatment (n = 8). **B-C.** Echocardiographic M-mode tracings and measurements of fractional shortening % (FS %) and ejection fraction % (EF %) 5 days after doxorubicin treatment (n = 8). **D.** Heart H&E staining of sham mice, doxorubicin-induced mice (DOX), and mice with APS pretreatment followed by doxorubicin treatment (DOX + APS) (n = 5, scale bar: 100 μM). **E.** TUNEL staining of apoptotic cells in the Sham, DOX and DOX + APS groups (n = 5, scale bar: 100 μM). **F.** Western blot and average data for LC3B II/I, Caspase-3, Bcl-2 in the sham, DOX, and DOX + APS groups (n = 5). (*P<0.05, **P<0.01, ***P<0.001).

After verifying the protective effect of APS *in vitro*, we then determined whether APS could preserve heart function *in vivo*. We generated a heart failure model using doxorubicin-treated C57BL/6 male mice. The body weight and heart weight/tibia length ratio decreased significantly after doxorubicin treatment for 5 days. Pretreatment with APS could significantly increase mouse body weight and heart weight/tibia length ratio (Figure 4A). Echocardiographic M-mode tracings and measurements were used to analyze heart function. Compared with sham mice, doxorubicin-treated mice exhibited decreased heart function as measured by the ejection fraction (EF%) and shortening index (FS%). APS treatment improved mouse heart function compared with that of doxorubicin treated mice. (Figure 4B-4C). As shown by H&E staining, doxorubicin-induced heart failure was associated with cardiac tissue disorder (Figure 4D). The cell apoptotic rate increased from 1.33±1.05% (Sham) to 22.28±8.39% (DOX) as demonstrated by TUNEL staining. Importantly, pretreatment with APS attenuated doxorubicin-induced cardiomyocyte apoptosis (11.53±3.54%) (Figure 4E). Western blot analysis also showed that APS suppressed doxorubicin-induced LC3B II/I and caspase-3 activation and promote Bcl-2 protein expression *in vivo* (Figure 4F).

### APS attenuates doxorubicin-induced heart injury by regulating the AMPK/mTOR pathway

**Figure 5:**
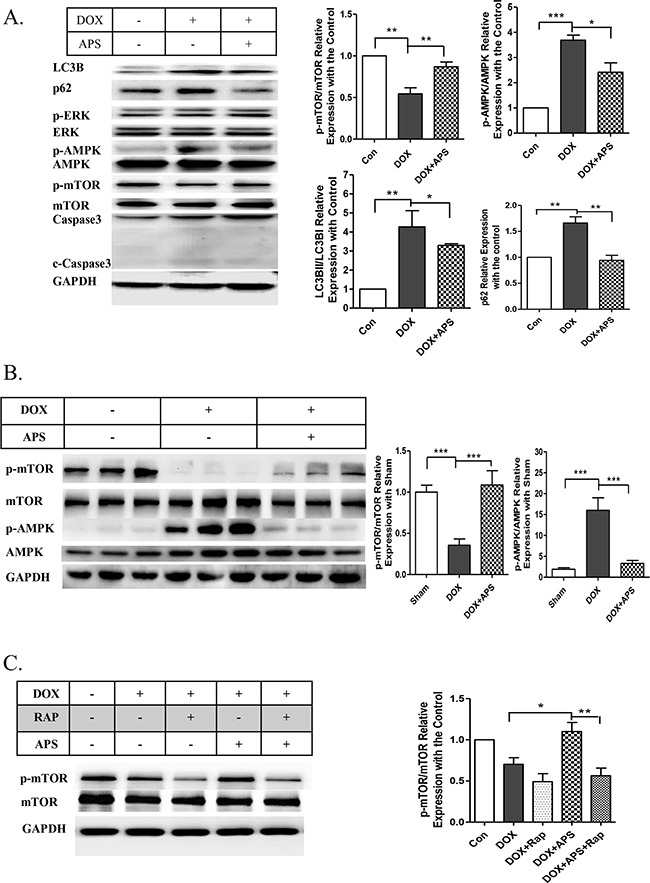
APS attenuates doxorubicin-induced heart injury by regulation of the phosphorylation of AMPK/mTOR **A.** APS (50 μg/ml) attenuateddoxorubicin (0.5 μM)-induced increased AMPK phosphorylation and decreased mTOR phosphorylation, as assayed by Western blots (n = 5). **B.** Western blot and average data for AMPK/mTOR phosphorylation in the hearts of sham, DOX, and DOX+APS pretreatment groups (n = 8). **C.** The protective effect of APS was reversed by the mTOR inhibitor rapamycin. The NRVMs were pretreated with 100 nM rapamycin for 1 h before APS (50 μg/ml) and DOX (0.5 μM) treatment. mTOR was analyzed by Western blots (n = 5). (*P<0.05, **P <0.01, ***P<0.001).

To further explore the protective effects of APS on doxorubicin-induced heart injury, autophagy-related signaling pathways were analyzed in response to doxorubicin treatment and APS pretreatment. The AMPK-dependent mTOR was determined to be responsible for the suppression of autophagy after APS pretreatment. According to the Western blot results, 0.5 μM doxorubicin treatment led to a significant decrease in mTOR phosphorylation, which in turn activated autophagy (Figure 5A). Interestingly, we found that AMPK, a vital regulator of the energy response and activator of mTOR, was substantially increased after doxorubicin treatment, and APS could inhibit its activation (Figure 5A). Similar results were obtained in the doxorubicin-induced heart failure mouse model (Figure 5B). Next, rapamycin was used to determine whether AMPK/mTOR regulates the protective role of APS in doxorubicin-induced cardiotoxicity. As shown in the Figure 5C, rapamycin significantly suppressed APS induced mTOR phosphorylation and reverse the protective effect. Taken together, these data suggest that APS could significantly restore autophagic flux and promote cell survival, which could be abrogated by the mTOR inhibitor rapamycin.

## DISCUSSION

Clinical use of doxorubicin as an effective anticancer chemotherapeutic agent for the treatment of solid tumors and hematologic malignancies is limited due to acute and chronic cardiotoxicity. *In vitro* and *in vivo* experiments have determined that doxorubicin could induce cardiomyocyte loss and heart dysfunction in a dose-dependent manner [24], which was confirmed by our study. However, the underlying mechanisms of doxorubicin cardiotoxicity are still unclear. Apoptosis induced by caspase-9/caspase-3 pathway activation and necrosis are believed to be involved in cardiomyocyte loss caused by a large dose of doxorubicin. However, high doses of doxorubicin are unrealistic, given current clinical use. Therefore, determining the role of autophagy under a low dose treatment is important to decrease its possible side effects.

Autophagy is an important cellular process that responds to various internal and external nutrients, but abnormal autophagy may cause multiple diseases, including cardiovascular diseases. Based on recent findings, it is highly possible that autophagy also acts as an important regulator in the pathogenesis of heart failure caused by doxorubicin treatment. In the present study, cell viability was significantly decreased in doxorubicin-treated cardiomyocytes and mice, with a concomitant significant elevation of LC3B II/I. To determine the effect of doxorubicin-induced cardiotoxicity on autophagy, we detected the autophagic flux in cardiomyocytes. According to a previous study, doxorubicin could impair the normal degradation of autophagosomes and halt autophagy [25]. In our study, we found that doxorubicin increased the formation of autophagosomes and blocked normal autophagic flux, which led to excessive accumulation of autophagosomes and autophagic cell death. Thus, autophagic death might play an important role in the cardiotoxicity caused by doxorubicin.

APS is a potent traditional medicine has been used to inhibit tumor growth, enhance immunity, and potently alleviate arterial inflammation [26, 27]. Several previous studies demonstrated that APS may be an effective treatment for heart diseases, such as myocardial hypertrophy and heart failure. Many clinical studies also indicated that APS could mitigate the side effects of chemotherapeutic drugs [17]. However, whether APS could protect cardiomyocyte from doxorubicin-induced cardiotoxicity was still not clear. In the present study, we found that APS significantly reduced doxorubicin-induced cardiomyocyte injury by restoring autophagic flux and autophagosome formation.

Previous studies revealed that doxorubicin treatment reduced the activation of mammalian target of rapamycin (mTOR) [28–30]. Interestingly, we found that APS treatment could dramatically decrease AMPK activation and promote mTOR activation. We hypothesized that AMPK/mTOR may be one of the key pathways in the protective effects of APS. Rapamycin is a classic reagent that induces autophagy by suppressing the phosphorylation of mTOR. According to our results, rapamycin could significantly reverse the protective effects of APS. Therefore, the data indicated APS could protect cardiomyocytes from doxorubicin-induced cardiotoxicity by suppressing phosphorylated AMPK, a negative regulator of mTOR phosphorylation, in turn reducing the level of autophagy.

In this study, pretreatment with APS could restore normal autophagic flux in doxorubicin-induced cardiotoxicity and improve heart function. These results suggest that APS may be a novel drug for the treatment of doxorubicin-induced cardiotoxicity.

There are several important issues that must be investigated before these findings can be applied to the clinic. Further studies are needed to assess the safety, efficacy and clinical application of APS. Second, APS affects various pathways; therefore, our study that focused only on its anti-autophagic properties is limited. Third, whether doxorubicin induces DNA damage should be determined. Therefore, p53 might be also involved in the doxorubicin-induced cardiotoxicity. Zhu et al (34) showed that acute doxorubicin cardiotoxicity is associated with p53-induced inhibition of mTOR. Thus, APS may not only inhibit AMPK activation but also regulate other upstream regulators to regulate mTOR and protect cardiomyocyte. Further studies are required to define the potential protective roles of APS in doxorubicin-induced cardiotoxicity.

In summary, our findings demonstrated that doxorubicin could induce cardiotoxicity by disturbing autophagic flux, which lead to excessive cell apoptosis. APS could restore normal autophagic flux, and ameliorate doxorubicin-induced cardiotoxicity by regulating the AMPK/mTOR pathway.

## MATERIALS AND METHODS

### Reagents

Antibodies against p62, Beclin1, ERK1/2, phosphorylated-ERK1/2, AMPK, phosphorylated-AMPK, mTOR, phosphorylated-mTOR, Bcl-2 and caspase-3 were purchased from Cell Signaling Technology (Danvers, MA, USA); GAPDH antibody was purchased from Santa Cruz Biotechnology (Santa Cruz, CA, USA); LC3B antibody was purchased from Sigma (St. Louis, MO, USA). Secondary antibodies directed against rabbit or goat were purchased from Cell Signaling Technology. Unless otherwise indicated, all chemicals were purchased from Sigma or Amresco.

### Preparation of APS

APS was purchased from the Shifeng Biological Co., Shanghai, China. APS was dissolved in PBS to generate a 10 mg/ml working solution and then diluted with DMEM culture medium at different concentrations for the following experimental treatments.

### Animals

Specific pathogen Free (SPF) male C57BL/6 mice (8 weeks old) were purchased from Beijing Vital River Laboratories and housed in SPF facilities. The mice were separated into three groups: sham, doxorubicin-treated, and doxorubicin-treated mice with APS. Sham mice were injected and orally administered saline solution. The doxorubicin-treated mice (DOX) were intraperitoneal injected with 20 mg/kg doxorubicin and orally administered APS or an equivalent volume of saline. The APS treatment group (DOX+APS) was pretreated with APS (1.5 g/kg/day) for 3 days by gavage, and this group was then intraperitoneal injected with 20 mg/kg doxorubicin. The APS treatment was continued for 3 days after doxorubicin injection [4, 23]. All the mice were euthanized 5 days after the initial injection of doxorubicin. All animal experiments conformed to the protocols approved by Animal Use and Care Committee of Beijing Hospital and the Guide for Care and Use of Laboratory Animals (NIH Publication #85-23, revised 1996).

### Echocardiography

Echocardiography was performed using Vevo 770 and Vevo 2100 (VisualSonics) instruments from Anzhen Hospital. According to the protocol, mice were lightly anesthetized with 1-1.5% isofluorane in oxygen until the heart rate stabilized to 400 to 500 beats per minute before echocardiography. Ejection fraction (EF)% and fraction shortening (FS)% were calculated with Vevo Analysis software as previously described [31].

### Histology

Tissues were processed by paraffin sectioning, and H&E staining was performed on the sections according to the manufacturer's protocol (Sigma-Aldrich).

### Terminal deoxynucleotidyl transferase-mediated dUTP nick-end labeling (TUNEL) and Hoechst 33342 staining of heart cryosections

Nuclear fragmentation was detected by TUNEL staining (Roche) and 10 mM Hoechst 33342 as previously described [23]. Cells in 10 randomly chosen fields from each dish were counted to semi-quantitatively determine the ratio of apoptotic nuclei. Each data point indicates results from 1600 to 2000 cells in 4 independent experiments [23].

### Isolation and culture of rat cardiomyocytes

Neonatal rat ventricular myocytes (NRVMs) were isolated from 1–3-day-old Sprague-Dawley rats using a combined trypsin and collagenase type II digestion method as previously described [23]. The cardiomyocytes were plated at a density of 6.6 × 10^4^ cells/cm^2^ in DMEM supplemented with 10% FBS and selected using 0.1 mM 5-bromo-2-deoxyuridine.

### Western blot analysis

Cell lysates were analyzed by 12% SDS-PAGE and transferred to PVDF membranes by wet transfer. The membranes were blocked with 5% milk for 1 h in room temperature and incubated with specific first antibodies (1:1000) overnight at 4°C. The primary antibodies were thoroughly washed with 5 washes of TBST (TBS containing 0.1% Tween 20), 10 min each; then, the membranes were incubated with horseradish peroxidase–conjugated secondary antibodies (1:3000) in TBST for 1 h at room temperature. Protein expression was then detected by ECL. Densitometry analysis was then performed with ImageJ software.

### GFP-LC3 adenovirus preparation and adenoviral transduction

The adenovirus GFP-LC3 was purchased from Hanbio Company of China. The cardiomyocytes were transfected with 30 M.O.I. of GFP-LC3 adenovirus for 24 h and then treated with DOX or DOX+APS for 24 h. The labeled autophagic vesicles were measured using ImageJ software and are shown as a percentage of the total number of GFP-LC3 dots.

### GFP and RFP tandemly tagged LC3 (tfLC3) assay

The method to evaluate tandem fluorescent LC3 puncta using the mRFP-GFP-LC3 plasmid was previously described [32]. H9c2 cells cultured on cover slips were transduced with the mRFP-GFP-LC3 plasmid for 24 h and then treated with DOX or DOX+APS for another 24 h. Then, the cells were viewed with an inverted fluorescence microscope. If there was a significant population of red-only signals, then the autophagosomes normally matured into autolysosomes (where the GFP is relatively unstable). If most puncta exhibited both red and green signals, autophagy was impaired at some step. Co-localization efficiency of mRFP with GFP signals of tfLC3 puncta was measured using ImageJ software and shown as the percentage of the total number of mRFP puncta.

### Statistical analysis

The data are expressed as the mean ± SEM. Student's *t*-test was used to compare two conditions, and one-way ANOVA with Bonferroni correction was used for multiple comparisons. Probability values less than 0.05 were considered significant.

## Abbreviations

AMPK: AMP-activated protein kinase
mTOR: mammalian target of rapamycin
TUNEL: terminal deoxynucleotidyl transferase (TdT)-mediated dUTP nick-end labeling
FBS: fetal bovine serum
PBS: phosphate buffered saline
GAPDH: glyceraldehyde-3-phosphate dehydrogenase
H&E: hematoxylin and eosin
PI3K: phosphoinositide-3-kinase
ERK: extracellular signal-regulated kinase.
